# Ubiquitin Is Not a Blood Biomarker of an Early Cognitive Decline in the Polish Elderly

**DOI:** 10.3390/cimb45030160

**Published:** 2023-03-16

**Authors:** Oliwia McFarlane, Mariusz Kozakiewicz, Milena Wojciechowska, Kornelia Kędziora-Kornatowska

**Affiliations:** 1 Department of Social and Medical Sciences, Ludwik Rydygier Collegium Medicum in Bydgoszcz, Nicolaus Copernicus University in Toruń, 85-089 Bydgoszcz, Poland; 2 Department of Geriatrics, Ludwik Rydygier Collegium Medicum in Bydgoszcz, Nicolaus Copernicus University in Toruń, 85-089 Bydgoszcz, Poland

**Keywords:** Alzheimer’s disease, biomarker, blood, dementia, mild cognitive impairment, ubiquitin

## Abstract

Together with development of new pharmaceutical interventions, as well as the introduction of the concept of initial dementia phase, the demand for early diagnosis has been growing. Research on potential blood biomarkers, amazingly attractive, mainly due to the facility of deriving the material, has provided ambiguous results throughout. The existence of an association between ubiquitin and Alzheimer’s disease pathology suggests that it could be a potential neurodegeneration biomarker. The present study aims to identify and assess the relationship between ubiquitin with regard to the adequacy as a biomarker of an initial dementia and cognitive decline in the elderly. Method: The study sample was composed of 230 participants: 109 women and 121 men aged 65 and older. The relationships of plasma ubiquitin levels with cognitive performance, gender, and age were analyzed. The assessments were performed in three groups of cognitive functioning level: cognitively normal, mild cognitive impairment, and mild dementia, of which the subjects were divided with the Mini-Mental State Examination (MMSE). Results: No significant disparities in plasma ubiquitin levels for various levels of cognitive functioning were identified. Significantly higher plasma ubiquitin levels in women were found in comparison to men. No significant differences were found in ubiquitin concentrations based on age. Results suggest that ubiquitin does not meet the requirements for qualification as a blood biomarker of an early cognitive decline. In order to thoroughly evaluate the potential of research on ubiquitin in connection to an early neurodegenerative process, further studies are needed.

## 1. Introduction

Together with development of new pharmaceutical interventions, as well as introducing the concept of the initial phase of neurodegenerative disorders, the demand for early diagnosis has been growing. Biological markers, which allow a diagnosis establishment at the early stages of the disease, are therefore much needed. Cerebrospinal fluid markers as well as functional imaging (such as Positron-Emission Tomography) can pose as factors facilitating diagnosis, but their usage is expensive and not easily accessible [1]. Implementing the neurodegenerative disease biomarkers (“cellular, biochemical or molecular alterations that are measurable in biological media such as human tissue, cells or fluids” [2]) would be much faster if they could be assayed in blood. Potential application of biomarkers includes monitoring therapy response, predicting clinical outcome, and measuring pathogenesis [3]. Optimal biomarker ought to be able to be assayed with a quick, safe diagnostic test and allow the detection of the condition in its preclinical phase [4]. An ideal diagnostic marker should also allow monitoring, should project condition progress, and should assess the efficacy of treatment methods—including potential treatment methods—during therapy or clinical trials.

Cerebrospinal fluid (CSF) has been the main focal point for following biochemical alterations associated with dementias, including AD. However, it is blood that is easily accessible and commonly used in tracking onset and progression of a variety of diseases involving peripheral organs. Approximately 500 mL of CSF are absorbed into the blood daily, making it a dependable source for monitoring cerebral disorders [5]. Moreover, it is inclined that the blood–brain barrier is compromised in an initial AD [6], possibly allowing brain-associated molecules to enter the blood stream. Research on potential plasma biomarkers, amazingly attractive, mainly due to the facility of deriving the material, has provided ambiguous results throughout. Some candidate biomarkers, including ubiquitin, demonstrate promising outcomes but have not yet been thoroughly assessed—especially in plasma [7].

Ubiquitin, first isolated from bovine thymus in 1975 by Goldstein et al. [8], is a small regulatory protein expressed in eukaryotic cells and associated with the cell cycle regulation [9]. It executes its various functions through conjugation to a vast spectrum of target proteins, which can result in a plethora of various alterations. The ubiquitin protein itself consists of 76 amino acids and has a molecular mass of about 8.6 kDa. Four genes in the human genome code for ubiquitin: UBB, UBC, UBA52, and RPS27A [10].

Ubiquitin is a crucial component of the ubiquitin–proteasome system (UPS), a main intracellular protein quality control and degradation system in eukaryotic cells [11]. UPS comprises many elements that incorporate the proteasome itself, ubiquitin hydrolases, E3 ubiquitin ligases, ubiquitin, and ubiquitin-like molecules. It selectively degrades targeted proteins by covalent conjugation to ubiquitin [12]. After the proteins have been connected to the ubiquitin chain, they are conducted to degradation via the UPS [12] or in the lysosome [13]. System malfunction is related to multiple neurological conditions including (but not limited to) Amyotrophic Lateral Sclerosis (ALS), Parkinson’s disease (PD), Transmissible Spongiform Encephalopathies (TSE), Huntington’s disease (HD), neuronal degeneration, or remodeling and regeneration after spinal cord injury (SCI) [14,15].

Multiple findings associate UPS dysfunction with both pathogenesis and progression of Alzheimer’s disease (AD) [16,17]. Protein quality control deficiency and protein misfolding or mishandling often cause neurodegenerative disease pathology [18,19,20]. AD is a protein-misfolding disease characterized by buildup of amyloid-beta (Aβ) peptides and hyperphosphorylated tau protein into plaques and neurofibrillary tangles, respectively [21]. Ubiquitin is also accumulated in the disease-typical structural alterations: ubiquitinated proteins are present in both neurofibrillary tangles and oligomeric Aβ plaques [22,23,24,25]. Proteasome dysfunction could therefore be the source event leading to the AD pathogenic cascade, the hypothesis according to which the accumulation of Aβ and tau is the original causative factor of AD. This suggests that dysfunction of the quality control mechanisms regulating protein breakdown might be—directly or indirectly—involved in the disease pathogenesis [12,13]. In a parallel manner, there is convincing evidence of a tight relationship between Aβ and UPS, thus playing an important role in AD progression [26]. The important role of the UPS in AD might be associated with impairments of the clearance of misfolded proteins preceding intracellular protein aggregation, cytotoxicity, and cell death [11,27]. Mutation in the UBB+1 gene that is related to the UPS triggers neuronal degeneration and is connected with both AD [28,29] and spatial memory impairment [30]. Defective proteasome activity is observed in the initial phase of AD in addition to synaptic dysfunction, and as the disease advances is linked with the buildup and aggregation of ubiquitinated proteins which leads up to tangle formation [31,32,33,34].

Animal studies have also brought data to support the association between both tau and amyloid-β with Small Ubiquitin-Like Modifier (SUMO) protein. This hypothesis was tested on 80 elderly people with dementia, 89 people with MCI, and 133 people without cognitive deficits, showing that plasma levels of SUMO1 are significantly elevated in patients with dementia compared to normal controls. SUMO1 levels correlated negatively with the Mini-Mental State Examination (MMSE), which suggests that elevated plasma SUMO1 can be associated with AD [35].

So far, very few studies have verified the associations between ubiquitin and cognitive performance; scarce available data are derived from reports on its CSF concentrations. The present study is one the first that aims to identify and assess the relationships between plasma ubiquitin and cognitive functioning of the elderly in the context of its usefulness as a biomarker of an early cognitive decline.

## 2. Materials and Methods

The study was conducted on the outpatients recruited from the Clinic of Geriatrics of the Nicolaus Copernicus University Collegium Medicum in Bydgoszcz, Poland. The study was conducted in accordance with the Declaration of Helsinki, and the protocol was approved by The Bioethic Committee of the Nicolaus Copernicus University Collegium Medicum UMK (no KB 505/2014). All the recruited subjects gave their written informed consent.

### 2.1. Participants

The study group was composed of 230 subjects (109 women and 121 men), aged 65 plus (age at sampling). Sampling followed strict exclusion criteria: (1) Geriatric Depression Scale 15-item version (GDS-15) > 5 points, (2) moderate or severe dementia, (3) brain stroke, and (4) other pathological states with possible severe impact on cognitive function.

To facilitate comparability, all analyses were performed in three age- and gender- matched groups of cognitive performance: cognitively normal (referred to as a control group), MCI, and mild dementia, of which the participants were divided with the Mini-Mental State Examination (MMSE) [36]. The tool had been administered by competent personnel. The total score of MMSE is 30 points, of which 30–28 points were indicative of cognitive norm, of which 27–24 points were regarded as MCI, and of which 23–20 points were considered mild dementia. Cognitively normal, regarded as control group, consisted of 71 respondents averaging 77.8 years old; the MCI group was composed of 85 patients, with the average age of 78.8; the mild dementia group was made of 74 participants, with the average age of 80.7 (Table 1). The global cognitive result was established as the MMSE score alone.

### 2.2. Biochemical Assessment

Blood samples were collected from each patient through vacuum venipuncture. Heparin plasma was stored in a low-temperature freezer located in the Clinic of Geriatrics of the Nicolaus Copernicus University Collegium Medicum in Bydgoszcz, Poland. Plasma concentrations of ubiquitin were assayed with the use of the ELISA immunoenzyme assay (CLOUD-CLONE CORP.; test sensitivity: <2.42 ng/mL). The used kit is a sandwich enzyme immunoassay for in vitro quantitative measurement of ubiquitin in serum, plasma, tissue homogenates, cell lysates, cell culture supernates, and other biological fluids. Prior to assay procedure, the samples were brought to room temperature (18–25 degrees Celsius).

### 2.3. Functional Assessment

Respondent data examination, encompassing selected clinical parameters (age, gender, socioeconomic variables, lifestyle factors, and health status), results of GDS-15 [37], Katz Index of Activities of Daily Living (ADL) [38], and Lawton Instrumental Activities of Daily Living Scale (IADL) [39], was performed.

### 2.4. Statistical Analyses

The main studied parameter regarding the level of cognitive performance was the MMSE test score. It was explored with regard to ubiquitin concentrations, with the application of suitable statistical methods, as outlined further. The functional evaluation (e.g., selected clinical variables, ADL, and IADL test results) was used to characterize the research group. Statistica 10.0 (StatSoft, Inc., Tulsa, OK, USA, 2016), R statistical packet (R Core Team, R Foundation for Statistical Computing, Vienna, Austria, 2016), and RStudio environment (RStudio Team, Integrated Development for R. RStudio, Inc., Boston, MA, USA, 2016) were used for all analyses. Based on the presence or not of a normal distribution of the values, data were expressed as mean and standard deviation or median with one and three quartiles. Normally distributed data were investigated with the use of the independent samples *t*-test or ANOVA with the RiR Tukey test, as appropriate. Not normally distributed data were evaluated with the use of the Mann–Whitney U test or the Kruskal–Wallis test, as appropriate. Correlations were analyzed with the use of the Spearman Rank correlation test for nonparametric distribution. *p*-value < 0.05 was considered to demonstrate statistical significance.

## 3. Results

### 3.1. Demographic and Functional Assessment

The study group was consistent in regard to socioeconomic, lifestyle, and health factors that carry the potential to influence cognitive functioning. The vast majority (78%) attained elementary or vocational education level. A total of 97% had a history of professional employment at some part of their lives, where 71% were physical workers or agriculturalists. A total of 76% of the respondents regarded their financial position as satisfactory. The sample was homogenous with regard to reproduction: 95% had children. A total of 76% of the patients admitted having performed some physical activity—mainly incidental activity integrated into daily routine—in the last 12 months. In spite of this fact, the study sample in general cannot be described as activity-oriented. Current participation in exercise or rehabilitation programs was declared by only 13% of the patients, while undertaking recreational or sport activities in the past was declared by 14% of the subjects. The ability to climb 1st floor was stated by 89% of the respondents, whereas only 13% declared the ability to swim a distance of 10 m. The majority of the sample viewed their health status as good; 18% were legally stated as disabled. A total of 87% showed normal thyroid function, with the remaining 13% qualified as having subclinical thyroid disease. A total of 73% of the sample had no vision problems. A total of 95% did not report history of depression treatment. A total of 84% declared their alcohol intake as less than few servings per year. A total of 97% of subjects were completely independent in basic ADLs.

### 3.2. Plasma Concentrations of Ubiquitin

No statistically significant differences in levels of ubiquitin between the cognitive performance groups were found (Table 2). Statistically significant differences were found in ubiquitin levels based on gender, with median ubiquitin concentrations being significantly higher in women than in men (Table 3). No statistically significant differences were found in ubiquitin levels based on age.

## 4. Discussion

The key issue in establishing preclinical diagnosis of dementia, critical for implementation of promising pharmaceutical interventions, is lack of an easy, cheap marker that would make a trusted indicator of an early cognitive deterioration. In spite of a decade of search, so far no single blood biomarker has been identified. Marker combinations that elevate diagnosis reliability, specificity, and sensitivity seem to be the most promising option. However, the current state of knowledge with conflicting results precludes their application in clinical practice [3].

It has been evident that the function of the UPS is disturbed in AD [16,31,40,41]. More and more studies indicate that there is a strong connection between UPS and beta-amyloid, with UPS having a significant impact on the amyloidogenic pathway of amyloid precursor protein (APP) processing that generates Aβ [17,26]. These discoveries in grasping the roles of the UPS in AD single out promising targets for prospective future medicinal interventions but also raise new concerns and research possibilities [26]. It is noteworthy that many UPS constituents, such as ubiquitin, are important determining factors of disease severity in AD. However, numerous ambiguities to be addressed in regard with the mechanisms by which it contributes to AD progression remain. In view of strong statistical evidence for existence of an association between ubiquitin and AD pathology, the aim of the undertaken study was to identify and assess the relationships between ubiquitin and cognitive performance of older adults in respect to its adequacy as a biomarker of an initial cognitive decline and dementia.

We found no significant differences in plasma ubiquitin concentrations between the study groups. In general, scarce available data suggest that ubiquitin CSF levels increase in AD patients [10]. There are reports on its elevation in cerebral cortex, detected by ELISA in brain homogenate in people with AD, which in turn correlates with the degree of neurofibrilary changes [42]. Findings from this study of a limited-size sample of under 20 patients with AD, other dementias, neurological disorders, and healthy controls report that the concentrations of ubiquitin were significantly elevated in subjects with AD in comparison to other groups. Additionally, the validity of ubiquitin as a diagnostic marker was regarded as equivalent to that of CSF Aβ42, tau, or the apolipoprotein Eε4 genotype considered individually. Moreover, a significant correlation was observed between ubiquitin, tau, and the ApoE4 allele, while correlation with Aβ42 was negative. Similarly, findings from an Indian study on the levels of Aβ42, total tau, and ubiquitin in CSF, measured with the use of the ELISA assays, suggest that high levels of ubiquitin are characteristic for AD [43]. Ubiquitin levels were significantly high (36.8 ± 4.34 ng/mL) in AD patients compared to those in non-AD (23.6 ± 2.32 ng/mL), neurological controls (19.8 ± 3.64 ng/mL), and healthy controls (13.2 ± 4.56 ng/mL). Our findings, inconsistent with the above, report lower concentrations of free ubiquitin in blood of MCI (17.6 ± 2.2 ng/mL) and mild dementia (17.2 ± 1.7 ng/mL) groups. It is possible that concentrations of ubiquitin in blood might be too low to be detected accurately enough. Conversely, ubiquitin levels reported by us in the control group (17.4 ± 2.3 ng/mL]) were higher than those of healthy Indian controls. Nonetheless, it has to be noted that due to the group differences in both studies, the possibility of direct result comparisons is limited; e.g., our control group, classified as cognitive norm, was selected with criteria which do not directly translate to full health.

Our findings seem to support the data obtained in a study aimed at exploring whether ubiquitin-activating enzyme E1, ubiquitin-conjugating enzyme E2, and ubiquitin or proteasome activity are affected in peripheral blood mononuclear cells (PBMC) of AD and MCI subjects compared to healthy controls. PBMCs were isolated from EDTA blood samples, and extracts were analyzed by Western Blot. Proteasome activity was measured with fluorogenic substrates. As observed, ubiquitin levels and proteasome activity remained unaffected in AD patients. Similarly, no modifications in enzyme expression or proteasome activity were reported in MCI subjects compared to healthy controls and AD patients [44].

Advanced age is the strongest predictor of AD. However, recent research [45,46] suggests the existence of sex disparities in regards with the disease in terms of both its prevalence and severity. Gender differences have also been noted in disease course, clinical manifestation, treatment response, and prognosis. It has been indicated that females are subject to a disproportionate burden in this regard. We have investigated gender differences in terms of free ubiquitin levels in blood and found significantly higher plasma ubiquitin levels in females in comparison to males. As outlined above, ubiquitination involves the attachment of ubiquitin to various target proteins resulting in regulation of their half-life, localization, activity, and conformation. Components of the ubiquitin system are often dysregulated, contributing to numerous diseases. Recently conducted analyses indicate that ubiquitin plays a pivotal part in multiple signaling and cell regulatory events in malignancies, including but not limited to gynecological [47] and breast cancer, which remain the most common carcinoma and second cause of premature death amongst women globally [48,49]. This may suggest the possibility of this gender-dependent elevation in concentrations of ubiquitin being related to the above findings. No significant differences were found in ubiquitin levels based on age.

To our best knowledge, this study was one of the first attempts to assess the relationships between this biochemical parameter and cognitive functioning. No significant differences in its concentrations between the study groups may suggest that ubiquitin does not constitute a good candidate for a potential plasma biomarker of an initial dementia in the elderly. These observations may, however, serve as the basis for further exploration.

However, limitations to this study have to be taken into consideration; only routine screening assessment of cognitive performance, emotional state, and functional status was executed. No results of further detailed tests or examinations, including brain imaging data, are currently available. Therefore, this study can be classified as preliminary research, with the need of its observations be confirmed through further work. Additionally, due to the fact that our study involved Polish elderly people exclusively, the results cannot be extrapolated to other populations. Moreover, the use of MMSE, which currently lacks the norms for under-educated individuals and consequently is not yet completely adjusted to the Polish senior population, could possibly contribute to research bias. In spite of the above-mentioned limitations, it is one of the first research attempts at exploring ubiquitin as a potential blood biomarker of an early cognitive decline and initial dementia in older adults. It could serve as a reference for future research on the ubiquitin content in other types of body fluids and the association with Alzheimer’s.

## 5. Conclusions

No statistically significant differences were found between ubiquitin and the level of cognitive performance. In accordance with the requirements for qualification as a blood biomarker of an early cognitive decline, it can be assumed that ubiquitin cannot aspire to a biomarker of an initial cognitive impairment in older adults. In order to thoroughly explore the prospects of research on small proteins with regard to an initial dementia and early cognitive decline, longitudinal cross-sectional studies aimed at further assessing their role in both the early and advanced MCI and dementia are needed.

## Figures and Tables

**Table 1:** Study group and cognitive functioning level (W—women, M—men, T—total).

Age Group	Norm			MCI			Mild Dementia			Total		
	W	M	T	W	M	T	W	M	T	W	M	T
65–74	13	13	26	17	14	31	7	13	20	37	40	77
75–84	12	15	27	11	15	26	12	14	26	35	44	79
85+	8	10	18	15	13	28	14	14	28	37	37	74
Total	33	38	71	43	42	85	33	41	74	109	121	230

**Table 2:** Plasma ubiquitin level (ng/mL) and cognitive performance (median [1 quartile–3 quartile]).

	Cognitive Functioning Level			ANOVA K-W *p* Level
	Norm	MCI	Mild Dementia	
**UBIQUITIN**	17.4 [16.4–18.7]	17.6 [16.8–19]	17.2 [16.4–18.1]	0.254

**Table 3:** Plasma ubiquitin level (ng/mL) and gender (median [1 quartile–3 quartile]).

	Gender		U M-W *p* Level
	Women	Men	
**UBIQUITIN**	17.56 [16.8–19.0]	17.18 [16.2–18.4]	0.032

## Data Availability

The raw data supporting the conclusions of this article will be made available by the authors, without undue reservation.
